# A Pilot Study of Cardiac MRI in Breast Cancer Survivors After Cardiotoxic Chemotherapy and Three-Dimensional Conformal Radiotherapy

**DOI:** 10.3389/fonc.2020.506739

**Published:** 2020-10-16

**Authors:** Carmen Bergom, Jason Rubenstein, J. Frank Wilson, Aimee Welsh, El-Sayed H. Ibrahim, Phillip Prior, Aronne M. Schottstaedt, Daniel Eastwood, Mei-Jie Zhang, Adam Currey, Lindsay Puckett, Jennifer L. Strande, Julie A. Bradley, Julia White

**Affiliations:** ^1^Department of Radiation Oncology, Washington University School of Medicine in St. Louis, St. Louis, MO, United States; ^2^Department of Radiation Oncology, Medical College of Wisconsin, Milwaukee, WI, United States; ^3^Department of Medicine, Division of Cardiology, Medical College of Wisconsin, Milwaukee, WI, United States; ^4^Department of Radiology, Medical College of Wisconsin, Milwaukee, WI, United States; ^5^Department of Medicine, Case Western Reserve University, Cleveland, OH, United States; ^6^Division of Biostatistics, Medical College of Wisconsin, Milwaukee, WI, United States; ^7^Department of Radiation Oncology, University of Florida College of Medicine, Jacksonville, FL, United States; ^8^Department of Radiation Oncology, James Cancer Hospital, The Ohio State University Comprehensive Cancer Center, Columbus, OH, United States

**Keywords:** 3D conformal radiation therapy, cardiotoxicity, cardiac MRI, radiation therapy, breast cancer

## Abstract

**Purpose/Objectives:**

Node-positive breast cancer patients often receive chemotherapy and regional nodal irradiation. The cardiotoxic effects of these treatments, however, may offset some of the survival benefit. Cardiac magnetic resonance (CMR) is an emerging modality to assess cardiac injury. This is a pilot trial assessing cardiac damage using CMR in patients who received anthracycline-based chemotherapy and three-dimensional conformal radiotherapy (3DCRT) regional nodal irradiation using heart constraints.

**Materials and Methods:**

Node-positive breast cancer patients (2000–2008) treated with anthracycline-based chemotherapy and 3DCRT regional nodal irradiation (including the internal mammary chain nodes) with heart ventricular constraints (V25 < 10%) were invited to participate. Cardiac tissues were contoured and analyzed separately for whole heart (pericardium) and for combined ventricles and left atrium (myocardium). CMR obtained ventricular function/dimensions, late gadolinium enhancement (LGE), global longitudinal strain (GLS), and extracellular volume fraction (ECV) as measures of cardiac injury and/or early fibrosis. CMR parameters were correlated with dose-volume constraints using Spearman correlations.

**Results:**

Fifteen left-sided and five right-sided patients underwent CMR. Median diagnosis age was 50 (32–77). No patients had baseline cardiac disease before regional nodal irradiation. Median time after 3DCRT was 8.3 years (5.2–14.4). Median left-sided mean heart dose (MHD) was 4.8 Gy (1.1–11.2) and V25 was 5.7% (0–12%). Median left ventricular ejection fraction (LVEF) was 63%. No abnormal LGE was observed. No correlations were seen between whole heart doses and LVEF, LV mass, GLS, or LV dimensions. Increasing ECV did not correlate with increased heart or ventricular doses. However, correlations between higher LV mass and ventricular mean dose, V10, and V25 were seen.

**Conclusion:**

At a median follow-up of 8.3 years, this cohort of node-positive breast cancer patients who received anthracycline-based chemotherapy and regional nodal irradiation had no clinically abnormal CMR findings. However, correlations between ventricular mean dose, V10, and V25 and LV mass were seen. Larger corroborating studies that include advanced techniques for measuring regional heart mechanics are warranted.

## Introduction

The use of regional nodal irradiation (RNI) for node positive breast cancer treatment after breast conserving surgery or mastectomy improves local control and survival (1, 2). However, the disease-specific survival advantage of RNI may be attenuated by higher non-breast cancer mortality (3, 4) secondary to cardiac causes (5–9). Patients with left-sided breast cancer receiving radiation have increased rates of major coronary events (9, 10) and cardiac mortality (7, 11). In addition, cardiac deaths (10, 12) correlate with extrapolated mean heart irradiation dose. There is an estimated approximately 4–16% relative increase in heart disease and/or major coronary events for each 1 Gy in mean heart dose received (9, 10, 13). Patients receiving internal mammary chain (IMC) nodal radiation (14) and patients treated with left-sided breast conserving therapy (15) also demonstrate higher late cardiac morbidity. In addition, many breast cancer patients who receive RNI also receive cardiotoxic anthracyclines as part of their chemotherapy (16). Anthracyclines have been shown to increase risk of systolic dysfunction and congestive heart failure as well as subclinical cardiac changes (17–19). Although this risk does not outweigh the survival benefit of anthracyclines (20), cardiac changes can be seen in survivors as far as 18 years or more from diagnosis (21). The interaction of anthracyclines and radiation on cardiovascular outcomes is not fully understood, but additional cardiac risk factors have been shown to increase the absolute risk of cardiac events after radiation therapy (9).

Many of the studies demonstrating increased levels of cardiac morbidity and mortality in breast cancer patients receiving radiation include mostly patients treated prior to the mid-1980s (4, 5, 8, 11). A number of recent breast cancer radiation techniques result in reduced radiation doses to the heart (22–25). Studies of more recent series of breast cancer patients have demonstrated lower excess cardiac mortality from radiotherapy (4, 5, 7, 26). Advances in radiotherapy such as three-dimensional conformal radiation therapy (3DCRT) have allowed quantification of heart irradiation doses and treatments that deliver lower doses of radiation to the heart. Further exploration is therefore warranted to assess the intuitive notion that modern 3DCRT techniques diminish damage to the heart, reduce adverse cardiac events, and improve overall survival.

Cardiac magnetic resonance imaging (CMR) is a powerful modality that allows for sensitive evaluation of cancer therapy-induced cardiac changes (27, 28). While CMR is not as widely used as echocardiograms, its utility is rapidly growing in cardiac research studies as an attractive modality. CMR is more reproducible than echocardiography (29), and CMR has been shown to be superior to echocardiography to identify cardiotoxicity in cancer survivors (30). Furthermore, CMR is not affected by acoustic window or geometric assumptions, and it is less dependent upon operator skills than echocardiography. CMR is also attractive due to its ability to acquire anatomical, functional, and perfusion information in one scanning period with one modality. CMR’s exquisite soft tissue contrast and spatial resolution (1–2 mm) may elucidate otherwise masked differences in cardiac parameters (31).

The purpose of this pilot study is to utilize CMR to examine cardiovascular function in women who received both anthracycline-based chemotherapy and RNI using 3DCRT and the use of heart constraints in treatment planning. This study also aims to explore whether CMR-demonstrated changes in perfusion, cardiac function, or cardiac anatomy correlate with the received radiation doses.

## Materials and Methods

This Institutional Review Board approved trial (NCT02348684) was conducted by screening a Medical College of Wisconsin database from 2000 to 2008 for lymph node-positive breast cancer patients treated with post-operative 3DCRT RNI with a pre-determined heart constraint (32). Clinicopathologic data were obtained from patient medical records. All patients received either left- or right-sided breast or chest wall irradiation, along with irradiation to the undissected axillary, supraclavicular, and IMC lymph nodes to doses of 45–50 Gy in 1.8–2 Gy fractions. The institutional cardiac dose constraints utilized during this time period required that less than 6% (ideal) and less than 10% (acceptable) (32) of the volume of the left ventricle received 25 Gy (V25), based on studies by Gagliardi and Gyenes (33–35). MHD was not constrained. All contours were recreated and/or verified for the study patients for consistency. Heart contours included (1) the whole heart as defined by pericardium, and (2) the ventricular volume as defined by both ventricles and the left atrium, excluding pericardium (32). Eligibility criteria were prior anthracycline chemotherapy, no cardiac disease (including heart failure, coronary heart disease, significant valvular disease, or cardiac event such as myocardial infarction) pre-breast cancer diagnosis, and no recurrent breast cancer. Patients unable to tolerate an MRI with contrast were excluded, as well as patients with active atrial fibrillation due to suboptimal CMR images in the setting of this arrhythmia. Eligible women were invited via letter to enroll in this IRB-approved trial. Fifteen left-sided patients and five right-sided patients were enrolled, for a total of twenty patients. CMR parameters evaluated included the following indicators of left ventricular (LV) and right ventricular (RV) function: ejection fraction (EF) and left ventricular mass index (LVMI). LV and RV dimensions were also obtained: LV end-diastolic volume index (LVEDVI), LV end-systolic volume index (LVESVI), RV end-diastolic volume index (RVEDVI), and RV end-systolic volume index (RVESVI). In addition, late gadolinium enhancement (LGE) and total LV myocardial extracellular volume (ECV) fraction were obtained as measures of cardiac scar and/or early cardiac fibrosis (36, 37). The LV was divided into three zones (basal, mid, and apical), and the short axis slices were set up visually to represent these areas. Planned analysis included correlation of CMR parameters with cardiac dose-volume constraints using Spearman correlations.

CMRs were obtained on a Verio 3T MRI scanner (Siemens Healthineers, Erlangen, Germany) with patients imaged in the supine position using commercially available RF transmitter/receiver coils. Geometric assessment of the LV was performed without contrast using a steady-state free precession (SSFP) cine sequence. Imaging parameters were: repetition time (TR) = 56.52 ms, echo time (TE) = 1.36 ms, asymmetric echo with factor 0.41, flip angle (FA) = 42°, field of view (FOV) = 252 mm^2^ × 300 mm^2^, matrix of 162 × 192 (in-plane pixel dimensions of 1.56 mm × 1.56 mm), slice thickness = 10 mm, receiver bandwidth (BW) = 1,240 Hz/px, parallel imaging using GRAPPA reconstruction (*R* = 2), and 25 cardiac phases. Gadolinium contrast agent (gadopentetate dimeglumine, *Magnevist*, Bayer Healthcare, Berlin, Germany) was administered at a rate of 0.2 mmol/kg and maximum dose of 20 mmol via peripheral IV. After a 10-min delay, LGE images were obtained for myocardial fibrosis assessment using a T1-weighted, segmented inversion-recovery (IR), fast gradient-echo (GRE) pulse sequence. Imaging parameters were: TR = 750 ms, TE = 1.94 ms, FA = 20°, FOV 340 mm^2^ × 265 mm^2^, matrix of 256 × 160, slice thickness = 10 mm, BW = 300 Hz/px, and no acceleration. Coverage of the entire LV was achieved by acquiring 6–8 short-axis (SAX) slices with 10-mm slice spacing, along with long axis images for cross validation. The same heart coverage with identical slice prescriptions were used for both cine and LGE imaging. Qualified cardiac MRI physicians (JR and AW) blinded to the patient identifying information, including dosimetric parameters and side of breast cancer, interpreted the CMR images.

Semi-automated quantification of LV volumes and myocardial mass was performed using CVI42 5.3.0 (Circle Cardiovascular Imaging, Calgary, Canada). Manual identification of the slice range to be segmented and the mitral valve annulus were performed. Optional corrections comprised manual contouring of epicardial or endocardial surfaces to restrict region-growth and adjusting blood sensitivity. LV volumes were quantified as the sum of short axis chamber volumes (2D area × slice thickness) measured during end-diastole (end diastolic volume, EDV) and end-systole (end systolic volume, ESV). EF was calculated as 100 × (EDV-ESV)/EDV. LV mass was calculated as the product of myocardial volume and specific gravity ([Epicardial EDV-Endocardial EDV] × 1.05). Global longitudinal strain (GLS) was calculated from short and long axis SSFP cine sequences utilizing the Tissue Tracking module of CVI42 5.3.0 (Circle Cardiovascular Imaging, Calgary, Canada).

Contrast-enhanced CMR images were analyzed to determine the amount of LGE versus normal LV myocardial volumes. Any hyperenhanced areas of LGE were manually planimetered by visual inspection in each SAX slice, including only regions that were fully enhanced and approximately ≥6 standard deviations (SD) above the mean signal of normal myocardium. Identical slices during cine imaging were compared to identify extent of blood pool and epicardial fat. Global ECV was obtained using the 3-3-5 MOLLI MyoMaps motion-corrected T1 map sequence (Siemens Healthineers, Erlangen, Germany) pre-contrast and 15 min post-contrast injection, and corrected with hematocrit obtained on the day of the CMR. Statistical analyses were performed using Graphpad Prism Version 7.0 (GraphPad Software, La Jolla, CA, United States) and SAS version 9.3 (SAS institute, Cary, NC, United States). Spearman’s correlations were calculated to determine correlation between heart dose and CMR parameters. Wilcoxon’s rank sum tests were used to compare CMR values in left- versus right-sided patients. *P* < 0.05 was considered significant.

## Results

Fifteen left-sided and five right-sided patients were enrolled in this study after obtaining informed consent, and the patients subsequently underwent CMR. The median age of the patients at diagnosis was 50 years (range 32–77). The median age at CMR was 60 years (range 40–83). The median time after 3DCRT was 8.3 years (range 5.7–14.4, Table 1). The presence of cardiac risk factors at the time of CMR is shown in Table 1. Patient characteristics were similar between groups (Table 1). All patients received doxorubicin or epirubicin as part of their chemotherapy. Fifteen patients received doxorubicin and cyclophosphamide, with thirteen patients receiving four cycles, one patient receiving three cycles, and one patient receiving two cycles. Three patients received six cycles of docetaxel, doxorubicin, and cyclophosphamide and two patients received docetaxol, epirubicin, and cyclophosphamide. Two patients also received trastuzumab (one left-sided patient and one right-sided patient) in addition to chemotherapy. Four patients had a ten pack-year or greater smoking history (three left-sided and one right-sided patient). Six patients had hypertension (five left-sided and one right-sided). Two left-sided patients had type II diabetes (Table 1). None of the patients had a history of clinical cardiac disease at the time of trial enrollment.

**TABLE 1:** Patient characteristics.

Median (range)	All patients (*N* = 20)	Left-sided (*N* = 15)	Right-sided (*N* = 5)
Age at diagnosis	50 (32–77)	49 (35–77)	52 (32–75)
Age at MRI	59 (40–84)	58 (41–84)	64 (40–80)
Follow-up (years)	8.3 (5.2–14.4)	8.2 (5.7–13.9)	8.5 (5.2–14.4)
Receipt of Trastuzumab	2	1	1
Smoking history ≥ 10 pack-year	4	3	1
Cardiovascular disease	0	0	0
Hypertension	6	5	1
Hyperlipidemia	6	6	0
Type II diabetes	2	2	0

*Patient characteristics and the presence of cardiovascular risk factors at the time of MRI are shown.*

**FIGURE 1:**
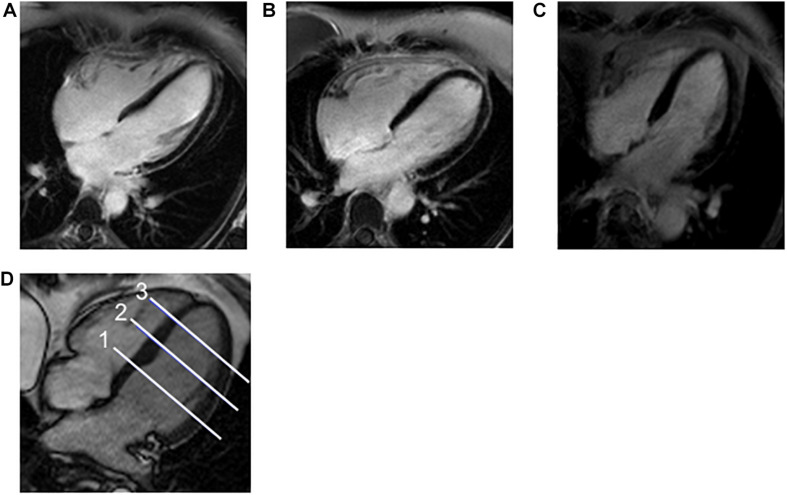
Example CMR Images and Location of Apex, Mid, and Base of Ventricles. **(A–C)** Representative four-chamber inversion recovery delayed enhancement images from three separate patients, without evidence of fibrosis. **(D)** A 4-channel steady-state free precession cine image demonstrating the usual position of the three short axis images that measure ECV for base (1), mid (2), and apex (3) of the left ventricle.

The heart dose-volume values for left- and right-sided patients are summarized in Table 2. Among right-sided patients, the median of the mean heart dose (MHD) was 0.6 Gy (0–1.0 Gy), and there was no volume of the heart receiving 5 Gy. Among left-sided patients, the median MHD was 4.8 Gy (1.0–11.2 Gy) and median heart V25 was 5.7% (0–12.4%); the maximum heart doses ranged from 11–54 Gy (Table 2). For left-sided patients, the median mean ventricular dose was 5.8 Gy (0.2–11.2 Gy) and median ventricular V25 was 5.2% (0–15.4%, Table 2). A summary of CMR values is provided in Table 3. Representative four-chamber inversion recovery delayed enhancement images from three separate patients, without evidence of fibrosis, are shown in Figure 1. The median LVEF was 63%. Pericardial thickness was normal in all patients (<4 mm), and no pericardial abnormalities were found. No first-pass perfusion abnormalities were seen. No late gadolinium enhancement was seen. Two patients had valvular abnormalities found on CMR (a left-sided patient with mild aortic regurgitation and a right-sided patient with mild mitral regurgitation).

**TABLE 2:** Patient cardiac dose-volume radiation parameters.

	Median	Mean	Range
**Left-sided patients**			
Mean heart dose	4.8 Gy	5.2 Gy	1.1–11.2 Gy
Max heart dose	51.9 Gy	49.2 Gy	11.4–54.4 Gy
Heart V5	16.4%	22.8%	0.2–63.5%
Heart V10	9.9%	14.5%	0–50.3%
Heart V25	5.7%	5.7%	0–12.4%
Heart V45	1.3%	1.3%	0–4.1%
Mean ventricular dose	5.8 Gy	5.6 Gy	0.2–11.2 Gy
Max ventricular dose	51.2 Gy	47.5 Gy	6.3–52.8 Gy
Ventricular V5	21.5%	28.3%	0–77.0%
Ventricular V10	12.1%	16.7%	0–55.1%
Ventricular V25	5.2%	5.9%	0–15.4%
Ventricular V45	0.7%	1.2%	0–3.5%
**Right-sided patients**			
Mean heart dose	0.6 Gy	0.6 Gy	0–0.1 Gy
Max heart dose	3.8 Gy	3.9 Gy	0–6.4 Gy
Heart V5	0 Gy	0 Gy	0 Gy
Heart V10	0 Gy	0 Gy	0 Gy
Heart V25	0 Gy	0 Gy	0 Gy
Heart V45	0 Gy	0 Gy	0 Gy
Mean ventricular dose	0.4 Gy	0.4 Gy	0–0.8 Gy
Max ventricular dose	2.4 Gy	2.0 Gy	0.2–3.3 Gy
Ventricular V5	0 Gy	0 Gy	0 Gy
Ventricular V10	0 Gy	0 Gy	0 Gy
Ventricular V25	0 Gy	0 Gy	0 Gy
Ventricular V45	0 Gy	0 Gy	0 Gy

*Abbreviations: V5, volume of the heart receiving 5 Gy; V10, volume of the heart receiving 10 Gy; V25, volume of the heart receiving 25 Gy; V45, volume of the heart receiving 45 Gy.*

**TABLE 3:** Cardiac MRI values of patients.

Median (range)	Normal range	All patients (*N* = 20)	Left-sided (*N* = 15)	Right-sided (*N* = 5)	*P* value (left vs. right)
LVEF	52–72%	63% (52–77%)	64% (52–77%)	61% (56–75%)	0.919
LVEDVI (ml/m^2^)	56–95	63 (46–83)	61 (46–74)	66% (56–83)	0.219
LVESVI (ml/m^2^)	14–34	23 (13–32)	22 (13–32)	26 (14–31)	0.500
LVMI (g/m^2^)	41–81	46 (32–56)	48 (32–56)	40 (38–50)	0.186
GLS	−22.1% to −15.9%	−14.6% (−17.8% to −11.1%)	−14.4% (−16.5% to −11.1%)	−15.6% (−17.8% to −12.9%)	0.161
ECV (total)	–	27% (23–34%)	27% (23–31%)	34% (24–34%)	0.119

*Abbreviations: LVEF, left ventricular ejection fraction; LVEDVI, left ventricular end-diastolic volume index; LVESVI, left ventricular end-systolic volume index; LVMI, left ventricular mass index; GLS, global longitudinal strain; ECV, extracellular volume fraction.*

Established abnormal values for ECV have not been universally agreed-upon (36, 38–40); however, some studies find that total LV myocardial ECV values correlated with higher likelihood of cardiac events and certain cardiac conditions (36, 40) and that ECV values correlated with myocardial fibrosis seen on biopsy (36). While some patients had total myocardial ECV values about 30%, these values were not higher in patients with left-sided tumors (Table 3), and higher ECV values did not correlated with increased heart (Table 4) or ventricular doses (Table 5). Most of the patients in this cohort had lower absolute GLS values than previously reported normal values (41), with 16/20 patients with lower absolute strain values (Table 3 and Supplementary Material). However, GLS did not correlate with doses of radiation received by the heart or ventricles (Tables 4 and 5). It is unclear whether these patients had lower absolute strain values due to anthracycline exposure or other cardiac risk factors.

**TABLE 4:** Correlation between CMR and cardiac radiation dose parameters in all patients, using whole heart dose-volume values.

Spearman correlation (*P* value)	Mean heart dose	Max heart dose	Heart V5	Heart V10	Heart V25
LVEF	0.044 (0.853)	0.020 (0.932)	0.108 (0.651)	0.105 (0.660)	0.082 (0.732)
LVEDVI	−0.091 (0.710)	−0.218 (0.371)	−0.198 (0.415)	−0.182 (0.455)	−0.013 (0.957)
LVESVI	0.033 (0.892)	−0.131 (0.594)	−0.165 (0.501)	−0.142 (0.563)	−0.004 (0.988)
LVMI	0.389 (0.099)	0.240 (0.323)	0.366 (0.122)	0.386 (0.103)	0.425 (0.070)
GLS	0.275 (0.241)	0.233 (0.324)	0.333 (0.152)	0.277 (0.238)	0.146 (0.540)
ECV (Total)	−0.359 (0.200)	−**0.467 (0.038)**	−0.388 (0.091)	−0.423 (0.063)	−0.422 (0.064)

*R-values and *P*-values are shown. Abbreviations: LVEF, left ventricular ejection fraction; LVEDVI, left ventricular end-diastolic volume index; LVESVI, left ventricular end-systolic volume index; LVMI, left ventricular mass index; GLS, global longitudinal strain; ECV, extracellular volume fraction. Bolded values indicate *P* < 0.05.*

**TABLE 5:** Correlation between CMR and cardiac radiation dose parameters in all patients, using ventricular dose-volume values.

Spearman correlation (*P* value)	Mean ventricular dose	Max ventricular dose	Ventricular V5	Ventricular V10	Ventricular V25
LVEF	−0.044 (0.429)	−0.020 (0.324)	0.108 (0.744)	0.105 (0.739)	0.082 (0.911)
LVEDVI	−0.091 (0.994)	−0.218 (0.353)	−0.198 (0.416)	−0.182 (0.613)	0.013 (0.893)
LVESVI	0.033 (0.596)	−0.131 (0.895)	−0.165 (0.501)	−0.142 (0.621)	−0.004 (0.750)
LVMI	**0.398 (0.012)**	0.240 (0.215)	0.366 (0.087)	**0.386 (0.027)**	**0.425 (0.016)**
GLS	0.237 (0.314)	0.222 (0.348)	0.370 (0.108)	0.300 (0.199)	0.126 (0.596)
ECV (total)	−0.411 (0.072)	−**0.454 (0.045)**	−0.379 (0.100)	−0.418 (0.067)	−**0.449 (0.047)**

*R-values and *P*-values are shown. Abbreviations: LVEF, left ventricular ejection fraction; LVEDVI, left ventricular end-diastolic volume index; LVESVI, left ventricular end-systolic volume index; LVMI, left ventricular mass index; GLS, global longitudinal strain; ECV, extracellular volume fraction. Bolded values indicate *P* < 0.05.*

LVM (or indexed LVM, LVMI) has been shown to be an independent risk factor for prediction of cardiovascular events (42). However, in this cohort, no abnormally elevated values were seen for LVMI or LV dimensions (Table 3). No abnormal CMR values were seen in the two patients who received trastuzumab. No correlations were seen between the MHD and max heart dose, heart V5, heart V10, heart V25, or the CMR parameters of LVEF, LVMI, or LV dimensions (Table 4). However, there were significant correlations between higher LVMI and the mean ventricular dose (*r* = 0.398, *P* = 0.012), the ventricular V10 (*r* = 0.386, *P* = 0.027), and the ventricular V25 (*r* = 0.425, *P* = 0.016, Table 5). Examination of correlations between basal, mid, and apical ECV values and heart or ventricular radiation doses also did not show any signification positive correlations (not shown). No correlations between increased total myocardial ECV measurements and higher heart doses (Table 4) or higher ventricular doses (Table 5) were seen.

## Discussion

A better understanding of the correlation between radiation doses to the heart and subclinical cardiac changes in breast cancer patients in modern series will be helpful to improve the therapeutic ratio of radiation therapy (43). This pilot study examined whether CMR could detect significant subclinical cardiac changes in women who received 3D conformal RNI (planned with heart constraints) and anthracyclines for node-positive breast cancer. With a median follow-up of 8.3 years in this cohort, CMR values were largely within normal limits. However, while the LVMI values were within normal limits (Table 3), there were significant positive correlations between LVMI and the ventricular mean dose, V10, and V25 (Table 5). In addition, increased total heart or ventricular doses were not statistically correlated with increased ECV values, a measure of extracellular volume that is increased in the setting of myocardial injury and an indicator of interstitial fibrosis (37). Correlations of unknown significance were seen between total LV ECV and maximum heart doses, as well as mid-LV ECV and ventricular doses, where lower ECV values correlated with higher radiation doses (Tables 4 and 5). It should be noted that the changes in LVMI without a corresponding change in ECV may be due to increased cardiomyocyte size causing hypertrophy, without or before reactive or replacement fibrosis, as has been seen post-radiation in some patients and preclinical models (44, 45). Total heart or ventricular radiation doses did not correlate with GLS values (Tables 4 and  5).

Radiation-induced cardiac events most commonly include pericarditis, myocardial fibrosis/scar, coronary artery disease, and valvular disease (19, 46). These deficits may be, at least in part, mediated by damage to the microvasculature, causing decreased coronary blood flow and resulting in diastolic dysfunction. This theory is supported by the fact that well-differentiated myocytes are relatively radioresistant. Anthracyclines, in contrast, are directly toxic to myocytes and thus are thought to cause cardiotoxicity possibly through a separate mechanism, which can potentiate the effects of radiation. Macrovascular injury may also contribute, as radiation promotes inflammation and oxidative damage–accelerating atherosclerosis (47). While anthracyclines significantly improve survival in breast cancer patients, notable cardiotoxic side effects can occur (17, 18, 48–50), which increase markedly with increasing dose (19). In this study, we specifically chose to examine potential radiation-induced changes to the heart in patients who not only received regional nodal irradiation, but also received anthracycline-based chemotherapy. In addition, two patients received trastuzumab, which can also cause cardiac injury (51). We saw no clinically abnormal CMR values in these patients, except GLS values (Table 3), which did not correlate with radiation doses (Table 4 and 5). However, the reproducibility of feature tracking can also be variable (52, 53). In addition, feature tracking is not as robust or sensitive as other standard strain measuring techniques, such as tagging, strain-encoding [SENC or fast-SENC (fSENC)] (54–59), or displacement-encoding with stimulated echoes (DENSE) (60). Future studies examining strain using CMR would ideally make use of these more advanced techniques. Especially relevant, recent studies illustrated the capabilities of fSENC for robust and detailed analysis of cardiac function and myocardial contractility pattern as fast as one slice per heartbeat. Such an approach would allow for evaluating cardiac function based on whole-heart strain analysis in a few seconds without the need for breath-holding or a contrast agent, which is of particular importance in cancer patients who may have difficulties with longer exams (56–59).

Previous studies have found trastuzumab to be cardiotoxic with 5% prevalence of cardiomyopathy when used as monotherapy and 10–15% prevalence when used with anthracyclines (51, 61). Despite receiving both radiation to the heart and cardiotoxic systemic therapy, our cohort of patients did not demonstrate significant abnormal CMR values for LVEF, LVEDVI, LVESVI, LVMI, or ECV. All patients in this study received IMC radiation as part of their RNI. Due to the proximity of IMC nodes to the heart, IMC irradiation can result in higher heart radiation exposure than radiation treatments omitting these regional lymph nodes. IMC nodal treatment has been controversial in practice (62, 63), but the recent MA.20 (64) and EORTC 22922 (65) studies demonstrated benefits of RNI that included treatment of the IMC nodes. Thus, rates of IMC nodal irradiation are likely to increase in the future.

To date nearly all studies evaluating outcomes by SPECT or other measures of cardiac injury have done so in breast cancer patients who did not have their radiation treatments planned with intent to treat IMC and with a heart constraint in place for dosimetric planning. Over the time period the patients in our study were treated, the heart constraint used for left-sided patients at our institution was ventricular V25 < 10% (32). This constraint results in higher MHD than currently accepted in clinical practice. In addition, the whole heart, including the pericardium and the ventricular tissue, were re-contoured on the radiation planning CT to determine received doses for the purposes of this study. In this cohort of patients receiving CMR with a ventricular dose constraint (but without a mean heart dose constraint), the mean heart doses were >5 Gy in 7/15 (47%) left-sided patients and >4 Gy in 10/15 (67%) left-sided patients, with mean heart doses >7.5 Gy in 3/15 (20%) left-sided patients. In right-sided patients, all had mean heart doses less than 0.7 Gy (Table 2). Even with left-sided patients receiving higher mean heart doses than recommended in current practice (mean heart doses <4 Gy when possible), at a median of over 8 years of follow-up, no perfusion defects or significant cardiac abnormalities were seen.

Perfusion changes have been detected in patients who recently received left-sided radiation therapy by using SPECT imaging, with changes correlating to the radiation treatment fields and with the percent of heart in the radiation fields (66–69). There is less data on long-term SPECT changes, although in one study a proportion of patients at 3 and 6 years had perfusion defects after radiation treatments in which the heart was not excluded from the treatment fields (70). Two prospective studies that used cardiac sparing techniques and excluded the entire heart from the radiation beams found no myocardial perfusion defects (71, 72). A recent study using echocardiogram with strain to examine cardiac function in breast cancer patients receiving contemporary radiation and cardiotoxicity systemic therapy did not reveal differences in strain post-radiation, although patients were examined only at 6 months after radiation (73). In this study, CMR was chosen for its potential to acquire anatomical, functional and perfusion information in one single scanning period, and for its increased spatial resolution (1–2 mm for CMR compared to 1.5 cm for SPECT). Our results are comparable to another study that used CMR to examine breast cancer patients treated with 3DCRT or IMRT up to 24 weeks of treatment. In that study, transient EF decreases were seen at 6 months on MRI, but this resolved by 24 months, and values for most parameters examined were in the normal range at 24 months, without the presence of wall motion abnormalities or late gadolinium enhancement (74).

In this pilot study, we examined women with long-term follow-up after completion of RNI and cardiotoxic chemotherapy using CMR, which can detect functional abnormalities and provide excellent spatial resolution. Taken together, our CMR data suggests that in this cohort there is no significant cardiac injury from receiving both RNI and cardiotoxic chemotherapy, despite relatively high mean heart doses received by a significant proportion of left-sided patients. It is important to note that patients included in this trial had no clinical evidence of cardiac disease at the time of treatment or prior to their study participation. Prior studies have demonstrated that cardiac events are more frequent after radiation in patients with baseline heart disease, thus these results are likely not applicable to this group (9).

In this study, positive correlations were found between ventricular dose volume parameters and LVMI. However, there was no evidence of increased ECV with increasing heart or ventricular radiation doses. It may be that even more refined regional cardiac analysis is necessary to study radiation-induced cardiac changes, as correlations may not be as evident when whole heart or ventricular doses are compared to global heart function. Indeed, the use of standardized cardiac heart substructure contours may allow more refined analysis of risk of cardiac disease based upon dose localization within the heart (75). For future studies, including advanced CMR techniques for studying regional heart mechanics (76) [e.g., MRI tagging (77), fSENC (54–59), or DENSE (60)] could reveal important information about myocardial regional contractility patterns that are expected to be affected at earlier timepoints after radiation, before reduction of global heart function and heart failure development. For global LV geometric measurements (LVEDV, LVEDVI, LVESV, LVESVI, LVEF and LVMI), only short-axis data was used. The inclusion of long-axis data and combining geometric data from multiple orientations in future studies would be expected to reduce the inherent geometric errors, potentially revealing significant changes that could have been masked in the current analysis.

Strengths of this study include the long median follow-up in patients who received anthracyclines and regional nodal irradiation, as well as receipt of radiation treatment using 3DCRT which allowed determination of cardiac and ventricular doses. However, this pilot study is limited by the small cohort of patients, as well as the lack of details regarding exercise capacity, detailed clinical symptoms, or cardiac biomarkers, although hypothesis-generating findings are seen with respect to ventricular radiation doses and LVMI. The CMR values were largely within normal limits despite a median MHD of >4 Gy for breast cancer patients enrolled in this study. This finding may reflect that in this population of patients without clinical cardiac disease there is a higher threshold for cardiac injury. Alternatively, this finding could mean that radiation therapy planned with cardiac constraints successfully limited partial heart injury, unlike prior studies evaluating cardiac injury from radiation delivered without constraints. It could also be that differences were present, but they were masked by the inherent uncertainties of in the estimation methods used. There is also the possibility of bias, as these 20 women self-selected from a cohort of women invited by letter to return for cardiac MRI. In addition, significant differences could have been present, but were masked by the inherent uncertainties in the estimation methods to obtain CMR values. For future studies, additional more sensitive analyses using CMR with multiple geometric views, regional strain analysis, and feature tracking may improve sensitivity to detect subclinical radiation changes. In addition, baseline CMR data was not available for this cohort of patients. However, the correlations seen between ventricular dose volume parameters and LVMI values are hypothesis-generating. Larger corroborating studies are warranted to further examine the utility of CMR in detection of therapy-induced heart disease in node positive breast cancer patients who receive both cardiotoxic chemotherapy and radiation.

## Data Availability Statement

All datasets generated for this study are included in the article/Supplementary Material.

## Ethics Statement

The studies involving human participants were reviewed and approved by Medical College of Wisconsin Institutional Review Board. The patients/participants provided their written informed consent to participate in this study.

## Author Contributions

JW, JAB, JR, and CB contributed to the conception and design of the study. CB, PP, JB, AW, AC, and JR collected the data. JR, CB, M-JZ, and DE performed statistical analyses. JR, AW, JLS, AC, and E-SHI interpreted the data. AMS and CB wrote initial drafts of the manuscript. JAB, JW, JFW, and LP wrote and edited sections of the manuscript. All authors contributed to manuscript revision, read, and approved the submitted version.

## Conflict of Interest

JAB has received a travel grant from Ion Beam Applications. The remaining authors declare that the research was conducted in the absence of any commercial or financial relationships that could be construed as a potential conflict of interest.
