# Supplementary material for: A Pilot Study of Cardiac MRI in Breast Cancer Survivors After Cardiotoxic Chemotherapy and Three-Dimensional Conformal Radiotherapy
Source: Front Oncol. 2020 Oct 16;10:506739. doi: 10.3389/fonc.2020.506739 (PMC7596658; doi:10.3389/fonc.2020.506739)
Supplement: Supplementary file 1 [file Table_1.pdf]

Supplemental Table 1. Patient Cardiac MRI Parameters

Patient Number	1	2	3	4	5	6	7	8	9	10	11	12	13	14	15	16	17	18	19	20
Mean heart dose (cGy)	551	924	465	184	683	168	342	344	709	554	458	788	480	1115	105	2	51	79	61	99
Max heart dose (cGy)	5205	5435	5195	5065	5345	5085	4950	5185	5125	5315	5205	5035	5425	5156	1145	24	321	377	635	607
V5-Whole heart (cGy)	37	57	16	6	34	3	12	11	21	15	14	29	20	64	0	0	0	0	0	0
V10-Whole heart (cGy)	14	33	10	4	22	1	7	8	16	10	10	22	9	50	0	0	0	0	0	0
V25-Whole heart (cGy)	3	8	6	2	8	1	3	4	12	7	6	11	4	10	0	0	0	0	0	0
V45-Whole heart (cGy)	0	2	3	0	1	0	0	1	0	4	2	2	1	1	0	0	0	0	0	0
Mean ventricle dose (cGy)	699	1115	532	247	20	190	486	422	696	578	597	1043	604	1112	117	2	35	59	40	77
Max ventricle dose (cGy)	4500	5280	5142	5070	4940	5160	4951	5128	4655	5215	5170	5120	5170	5111	625	23	243	255	159	332
V5-Ventricle (cGy)	57	77	22	8	25	4	18	15	22	17	19	39	33	70	0	0	0	0	0	0
V10-Ventricle (cGy)	18	42	12	6	11	2	11	10	16	10	14	30	14	55	0	0	0	0	0	0
V25-Ventricle (cGy)	2	9	6	3	3	1	5	5	12	7	8	15	5	8	0	0	0	0	0	0
V45-Ventricle (cGy)	0	2	2	1	1	0	1	1	0	3	2	3	1	1	0	0	0	0	0	0
LVEF (%)	72.4	52.1	76.9	54.0	72.9	56.2	67.2	64.2	63.8	57.2	69.3	57.2	59.3	63.9	62.2	71.3	74.5	55.5	60.8	55.7
RVEF (%)	59.4	53.7	61.8	47.1	59.2	49.1	53.9	54.0	61.4	65.5	62.4	66.1	53.7	63.9	60.3	55.1	58.1	59.9	63.8	53.8
LVEDV (ml)	120.2	85.1	100.9	109.0	104.1	89.1	111.5	115.0	133.7	129.1	146.6	123.9	121.3	98.1	97.3	143.4	121.5	123.9	114.8	123.4
LVEDVI (ml/m2)	61.0	47.1	54.5	61.8	54.9	45.9	55.9	65.6	71.9	74.1	65.7	67.3	67.7		57.9	82.9	56.4	70.3	65.7	62.5
LVESV (ml)	33.2	40.8	23.4	50.0	28.2	39.0	36.6	41.1	48.4	55.3	44.9	53.1	49.4	35.4	36.8	41.1	30.9	55.1	45.0	54.7
LVESVI (ml/m2)	16.8	22.6	12.6	28.4	14.9	20.1	18.4	23.5	26.1	31.7	20.1	28.9	27.6		21.9	23.8	14.4	31.3	25.8	27.7
LV Mass (g)	96.5	83.9	77.6	99.2	72.6	62.5	86.2	79.7	103.5	85.4	120.2	95.0	90.1	66.3	70.4	79.0	108.1	69.8	66.6	79.8
LVMI (g/m2)	49.0	46.4	41.9	56.2	38.3	32.2	43.3	45.5	55.7	49.0	53.8	51.6	50.3		41.8	45.7	50.2	39.6	38.1	40.4
RVEDV (ml)	90.8	76.1	99.5	101.6	90.6	103.2	148.5	84.4	105.5	113.1	132.2	73.3	104.1	81.3	65.4	119.2	136.5	128.3	87.4	107.7
RVEDVI (ml/m2)	46.1	42.1	53.8	57.6	47.8	53.2	74.5	48.1	56.8	64.8	59.2	39.8	58.1		38.9	69.0	63.3	72.8	50.0	54.6
RVESV (ml)	36.9	35.2	38.0	53.7	37.0	52.6	68.4	38.8	40.7	39.1	49.7	24.8	48.3	29.3	25.9	53.5	57.2	51.5	31.6	49.7
RVESVI (ml/m2)	18.7	19.5	20.5	30.5	19.5	27.1	34.3	22.1	21.9	22.4	22.3	26.3	26.9		15.4	30.9	26.6	29.2	18.1	29.4
GLS (%)	-13.9	-11.1	-14.6	-14.6	-14.1	-13.4	-13.2	-13.7	-15.4	-16.2	-16.5	-14.2	-14.4	-14.4	-14.5	-14.7	-17.8	-12.9	-15.6	-15.9
Total ECV (%)	27.1	23.2	23.5	27.3	28.4	26.2	31.2	25.1	23.2	27.7	26.5	28.9	27.8	27.4	28.2	28.5	24.0	33.7	29.3	33.7

Abbreviations: LVEF, left ventricular ejection fraction; RVEF, right ventricular ejection fraction; LVEDV, left ventricular end-diastolic volume; LVEDVI, left ventricular end-diastolic volume index; LVESV, left ventricular end-systolic volume; LVESVI, left ventricular end-systolic volume index; LVM, left ventricular mass; LVMI, left ventricular mass index; RVEDV, right ventricular end-diastolic volume; RVEDVI, right ventricular end-diastolic volume index; RVESV, right ventricular end-systolic volume; RVESVI, right ventricular end-systolic volume index; GLS, global longitudinal strain; ECV, extracellular volume fraction.
